# Preventive residual insecticide applications successfully controlled *Aedes aegypti* in Yucatan, Mexico

**DOI:** 10.1038/s41598-022-26577-1

**Published:** 2022-12-20

**Authors:** Gonzalo M. Vazquez-Prokopec, Azael Che-Mendoza, Oscar D. Kirstein, Wilberth Bibiano-Marin, Gabriela González-Olvera, Anuar Medina-Barreiro, Hector Gomez-Dantes, Norma Pavia-Ruz, Pablo Manrique-Saide

**Affiliations:** 1 Department of Environmental Sciences, Mathematics and Science Center, Emory University, 400 Dowman Drive Ste: E530, Atlanta, GA 30322 USA; 2 Unidad Colaborativa Para Bioensayos Entomologicos, Universidad Autonoma de Yucatan, Mérida, Mexico; 3 National Institute of Public Health, INSP, Cuernavaca, Mexico; 4 Centro de Investigaciones Regionales, Autonomous University of Yucatan, Mérida, Mexico

**Keywords:** Viral infection, Preclinical research

## Abstract

Insecticide-based approaches remain a key pillar for *Aedes*-borne virus (ABV, dengue, chikungunya, Zika) control, yet they are challenged by the limited effect of traditional outdoor insecticide campaigns responding to reported arboviral cases and by the emergence of insecticide resistance in mosquitoes. A three-arm Phase II unblinded entomological cluster randomized trial was conducted in Merida, Yucatan State, Mexico, to quantify the entomological impact of targeted indoor residual spraying (TIRS, application of residual insecticides in *Ae. aegypti* indoor resting sites) applied preventively 2 months before the beginning of the arbovirus transmission season. Trial arms involved the use of two insecticides with unrelated modes of action (Actellic 300CS, pirimiphos-methyl, and SumiShield 50WG, clothianidin) and a control arm where TIRS was not applied. Entomological impact was quantified by Prokopack adult collections performed indoors during 10 min per house. Regardless of the insecticide, conducting a preventive TIRS application led to significant reductions in indoor *Ae. aegypti* densities, which were maintained at the same levels as in the low arbovirus transmission period (Actellic 300CS reduced *Ae. aegypti* density up to 8 months, whereas SumiShield 50WG up to 6 months). The proportional reduction in *Ae. aegypti* abundance in treatment houses compared to control houses was 50–70% for Actellic 300CS and 43–63% for SumiShield 50WG. Total operational costs including insecticide ranged from US$4.2 to US$10.5 per house, depending on the insecticide cost. Conducting preventive residual insecticide applications can maintain *Ae. aegypti* densities at low levels year-round with important implications for preventing ABVs in the Americas and beyond.

## Introduction

The rapid propagation of dengue and other *Aedes*-borne viruses (ABVs; e.g., chikungunya, Zika) throughout the Americas, particularly in cities with well-established and adapted *Aedes aegypti* populations^1^, has evidenced the difficulties in responding and preventing arbovirus outbreaks. A myriad of factors have explained this rapid arboviral range expansion and increased transmission intensity, including *Aedes aegypti* mosquito expansion^2^, rapid and unplanned urbanization^3^, the disproportionate contribution of inapparent infections to transmission^4,5^ and human mobility patterns^6^. An additional challenge for effective ABV control stems from the remarkable paucity of evidence about the epidemiological impact of current vector control methods^7,8^.

The World Health Organization (WHO) guidelines for dengue prevention and control^9^, operationalized in the Americas by the Pan-American Health Organization (PAHO), emphasize the need for integrated vector management (IVM) approaches to control *Ae. aegypti*^10^ and prevent ABV outbreaks. IVM centers in the integration of tools to attack multiple risk factors of human-mosquito contact. Traditional methods such as environmental management (reduction or elimination of potential larval habitats such as plastic containers and unused yard debris or fixing large water-holding containers) and chemical control (e.g., use of chemicals to kill larvae such as insect growth regulators or Bti, and application of ultra-low volume or thermal fogging of insecticides to kill adult mosquitoes) have been and continue to be key pillars of the plan^10,11^. In most countries, the operationalization of IVM has encountered multiple roadblocks, including saturated and under-resourced health services^12^, the emergence of insecticide resistance^13^, and limited practical guidance on how and when to deploy vector control interventions in different epidemiological settings^11^. The Global Vector Control Response 2017–2030 from the WHO, which provides a new strategy to strengthen vector control worldwide, now emphasizes the need for locally adapted vector control as a paradigm for incorporating existing and novel approaches within IVM plans^11,14^.

Results from recent clinical trials evaluating *Wolbachia* releases^15^ or house screening^16^ and from mathematical models^17,18^ shown the important public health gains of conducting interventions that impact *Ae. aegypti* continuously and preventively, rather than reactively to increases in transmission intensity. The impressive success of perifocal spraying of DDT to eradicate *Ae. aegypti* from the Americas relied on long-lasting residual insecticides^19^. Similarly, the application of indoor residual spraying (IRS, the broadcast application of long-lasting insecticides indoors, primarily on walls, ceiling, and other surfaces) against malaria vectors also led to a significant reduction in dengue burden both in British Guyana and Cayman Islands^20–22^. Despite this promising evidence, preventive *Ae. aegypti* control is not yet considered as an integral part of any IVM program in the Americas, likely due to the limited existing evidence about its cost and efficacy compared to reactive interventions.

An improvement to IRS, that takes into consideration the known preferential resting of *Ae. aegypti* indoors at heights lower than 1.5 m^23^ (termed targeted indoor residual spraying, TIRS), consists of the selective application of residual insecticides on exposed low walls [< 1.5 m], under furniture and on dark surfaces^24^. TIRS is considered a rational alternative to IRS, given it reduces the time and amount of insecticide used to spray a premise with no apparent loss in efficacy^24,25^. In Cairns, Australia, TIRS using alpha-cypermethrin and deployed in premises identified by contact tracing as potential exposure sites during an outbreak reduced the probability of future DENV transmission by 86–96% as compared to unsprayed premises^26^, whereas entomological cluster-randomized control trials (CRCT) conducted in Mexico showed sustained reductions in *Ae. aegypti* abundance of up to 70% when the carbamate insecticide bendiocarb was used^27^. The recent availability of new insecticide molecules with long-lasting residual power (> 6 months) with alternative modes of action that counter the emergence of pyrethroid resistance^28^, could lead to a change in the implementation of TIRS.

Modeling studies predict that, when insecticide residual power lasts at least 5 months, TIRS epidemiological efficacy is highest if interventions are conducted prior to the beginning of the transmission season^17,18,29^. Preventive TIRS applications (i.e., pre-season intervention delivery) may offset the low coverage that may be achieved if intervention is implemented reactively to symptomatic reported cases^18^ potentially leading to increases in coverage, intervention efficiency and entomological impact. Here we present results from a three-arm unblinded entomological CRCT evaluating the efficacy of preventive TIRS application of two long-lasting insecticides on *Ae. aegypti*, provide information about costs of the method when implemented by public health agencies, and describe the community response to the pre-season insecticide application.

## Methods

### Trial design

**Figure 1:**
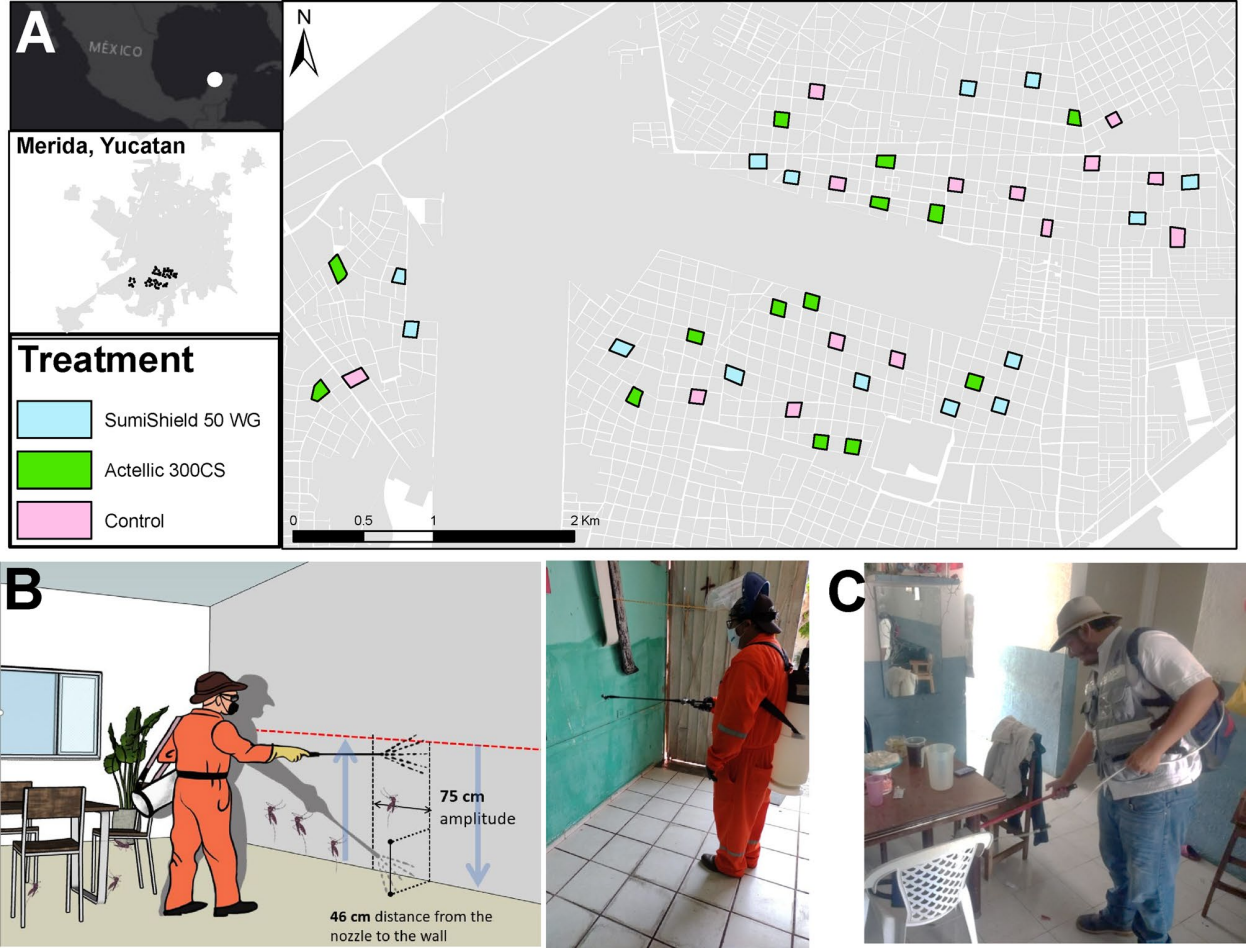
Overall trial design. (**A**) Distribution of clusters randomly selected for the CRCT and study arms (colors) in the city of Merida, Yucatan, Mexico (map inset). Images show the implementation of TIRS during the study (**B**) and an entomologist using Prokopack aspirators (**C**).

An unblinded three-arm CRCT in which entire city blocks were used as clusters (14 blocks per arm, 42 blocks in total) and were randomly assigned to receive TIRS or not (reactive space spraying by the MOH, considered as the control) was conducted in the city of Merida, Yucatan, Mexico. Merida is endemic for *Ae. aegypti* and ABVs^30^ and the Collaborative Unit of Entomological Bioassays dependent of the Autonomous University of Yucatan (UCBE-UADY) has established a reputable infrastructure for conducting trials evaluating vector control tools^16,25,27^. The study occurred in an area identified as a hotspot of ABV transmission within Merida^30^. Clusters had between 19 and 23 houses and were separated by at least one city block (Fig. 1). The three arms for the trial involved: a) control (no insecticide applied by the research team but reactive peridomestic malathion spraying by the MOH in response to symptomatic ABVs); b) Actellic 300CS (active ingredient, a.i., pirimiphos-methyl, Syngenta); c) SumiShield 50WG (a.i., clothianidin, Sumitomo Chemical Co. Ltd.). These two next-generation residual insecticides have proven efficacy against *Anopheles* spp.^31,32^ and were efficacious against *Ae. aegypti* on Phase II using WHO cone bioassays on different treated surfaces^33^.

The study was carried out during 2018–2019 and included preventive TIRS applied before the typical ABV transmission season (which spans from July to December^1^). A pre-intervention entomological survey (2 months prior to TIRS, April 10–16 2018) was followed by pre-season TIRS (June 16–29 2018) and monthly entomological surveys for 8 months post-TIRS to cover the typical transmission season (July-December 2018) and 2 months after the arbovirus season (January–February 2019). We conducted the baseline 2 months prior to spraying to determine whether our study arms were balanced with regard to *Ae. aegypti* density (2 months gave us enough time to enroll new houses should arms be unbalanced). Since both arms were balanced, we did not require any further enrollment. Additionally, we did not use any information from the baseline to inform any analysis of preventive TIRS. Entomological surveys were conducted using Prokopack aspirators^34^ indoors on a random sub-sample of 10 houses per cluster to quantify indoor *Ae. aegypti* density and presence^27^. Briefly, each house was visited by two field collectors, one in charge of interviewing householders and the other of conducting Prokopack aspirations. Prokopack collections lasted for a total of 10-min per house and involved the systematic collection of mosquitoes in all available rooms in the house (closed rooms were excluded), including bedrooms, bathrooms, living rooms and kitchens. On each room, the collector walked with the Prokopack aspirator turned on, waving the device in dark areas below 1.5 m where *Ae. aegypti* is known to rest^23^ (under beds, furniture, near dark objects, clothing or other apparel used by people). Any flying insects detected by the collector (using a headlamp) were also collected by aiming the aspirators at them. A timer was used to make sure collections ended at the 10-min mark. Collection cups, individualized by house, were placed in a cooler and taken to the entomological laboratory at UCBE-UADY at the end of the collection day for species identification, sorting of bloodfed *Ae. aegypti* females, and data entry. Table S1 shows the timing of each date with regards to the ABV season in Merida. Three months after spraying (July 2018), a survey to assess community concerns and acceptance of TIRS was applied to 150 households, split between intervention arms.

### Ethics statement

All methods were carried out in accordance with relevant guidelines and regulations. All study protocols were approved by Emory University Institutional Review Board (IRB00110234) as well as the Servicios de Salud de Yucatan. Written informed consent was obtained from the household owner and houses who did not consent to the intervention were noted and not sprayed or visited in post-intervention entomological surveys. Since this was an entomological CRCT (non-clinical), no registration on ClinicalTrials.gov or WHO trials databases was pursued.

### TIRS intervention

Insecticides had different presentations. Sumishield 50 WG was formulated as 150 g sachets containing 50% w/w clothianidin in a water dispersible granule. Actellic 300CS was formulated as 833 ml bottles of capsule suspension containing 28.2% pirimiphos-methyl. We followed WHO/PAHO guidelines for TIRS implementation^24^. Briefly, both insecticides were mixed in 7.5 L of water and applied with a manual compression sprayer IK-Vector Control Super (Goizper Group, Antzuola, Spain) with a 8002EVP nozzle and a Goizper low-pressure control flow valve (output pressure 1.5 bar) to provide a flow rate of 580 ml per minute (± 5%), and a target dose of 300 and 1000 mg a.i./m^2^ for SumiShield 50WG and Actellic 300CS, respectively. From June 17 to 26 a total of 500 houses (248 for Actellic 300CS: 252 for SumiShield 50WG) were intervened. Six teams with 3 persons each were assigned different clusters for conducting participant and household enrollment (including informed consent), community engagement, and spraying. World Health Organization cone bioassays were conducted monthly up to 8 months post-intervention on ten houses receiving each insecticide using laboratory-reared susceptible *Ae. aegypti* females (Rockefeller strain) and estimating acute (2 h after exposure) and delayed (1 day to 7 days after exposure) mortality. Susceptible females were used because the goal of the bioassays was to quantify insecticide residual activity, not the impact of insecticides on field populations. A total of 20 females were placed on each cone, with four replicate cones conducted per house per month. Standard *Ae. aegypti* ovitraps^35^ were placed in the front door of 10 houses in treatment clusters and ninety female *Ae. aegypti* mosquitos derived from the collected eggs were used to tested for susceptibility to different a.i. using the CDC bottle bioassay at the recommended diagnostic dose (permethrin = 15 µg/bottle; deltamethrin = 10 µg/bottle; chlorpyrifos 50 µg/bottle) and assessed for knock-down and 24 h mortality. A total of 30 females were place per bottle, and three replicate bottles were used per insecticide. At the time of the study, no diagnostic doses were established for pirimiphos-methyl or clothianidin in *Ae. aegypti*. We used chlorpyrifos as an a.i. with potential cross-resistance with pirimiphos-methyl, since both are organophosphates and chlorpyrifos was used by the MOH to control *Ae. aegypti* at the time of the study.

### Analysis plan

The two endpoints for the trial were the density (number of *Ae. aegypti*) per house after a 10-min Prokopack aspiration indoors and number of bloodfed female *Ae. aegypti* (a closer proxy to transmission risk than adult mosquito density) per house. Generalized linear mixed models (GLMM) with a negative-binomial function were applied to quantify the efficacy of each treatment compared to the control arm on each sampling date. We ran an overall model, including all post-intervention surveys, which included a random intercept for the survey month and another for the cluster ID. The finding of a significant overall model provided the opportunity to evaluate the efficacy of each intervention by survey date, in which case only cluster ID was used as a random intercept to nest house sub-samples to the cluster level. The overall and per-survey date intervention efficacy in reducing *Ae. aegypti* density was calculated as *E* = *1-IRR*, where IRR is the Incidence Risk Ratio calculated from the negative-binomial GLMM^27^. Costs of each component (provided by Yucatan MOH) were used to estimate the per-house cost of TIRS implementation. Since SumiShield 50WG is not commercialized in Mexico, we used the price of Actellic 300CS to the MOH as a reference, and then considered an alternative costing scenario considering the insecticide prices for global malaria control programs.

## Results

**Figure 2:**
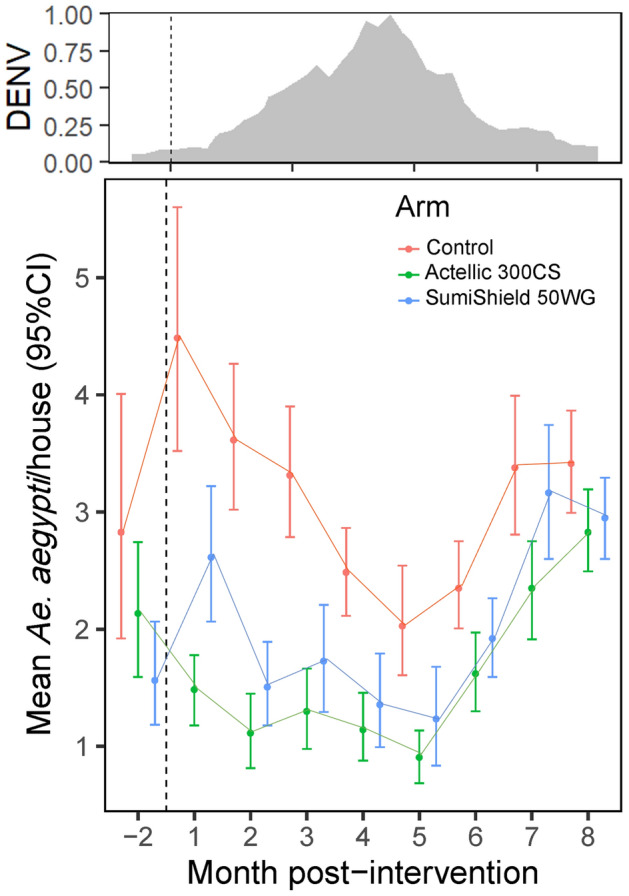
Impact of pre-season TIRS on *Ae. aegypti* density. Estimated mean (± 95%CI) number of *Ae. aegypti* adults per house at baseline (April) and 1–8 months (July 2019-January 2020) post-TIRS application of two long-lasting residual insecticide formulations in Merida, Mexico. TIRS was conducted in June (dashed vertical line), prior to the regular arbovirus transmission season. Top panel shows the standardized incidence of dengue from 1991–2001 in Yucatan, with values closer to 1 indicating periods of high transmission and values closer to 0 of low transmission.

TIRS averaged 9.4 ± 4.8 min per house and 1083 ± 709 ml of insecticide per house (Table S2). Kitchens and bathrooms were not treated, leading to lower number of rooms treated than available (Table S2). A total 3780 house entomological surveys were conducted throughout the study. The average (± Standard Deviation) number of *Ae. aegypti* per house during the 8-months post-TIRS was 3.13 (3.62) in the control arm, versus 1.59 (2.09) for Actellic 300CS and 2.06 (2.80) SumiShield 50WG. Adult *Ae. aegypti* density in the control arm increased rapidly as the ABV transmission season progressed, peaking at an average (± 95%CI) of 4.5 ± 3.5–5.5 adults per house on July (1 month post-intervention, MPI). Both pre-season TIRS treatments showed a dramatic reduction in adult *Ae. aegypti* density per house compared to the control (Fig. 2). Interestingly, adult *Ae. aegypti* density was maintained at levels lower than the pre-season density for up to 5 MPI (past the seasonal ABV transmission peak) in both TIRS arms (Fig. 2). A similar temporal trend was observed for the density of bloodfed females (Fig. S1). Only 18 dengue cases were reported in the south of Merida in 2018, none of them within any of the treatment clusters (Fig. S2). The low number of cases led the MOH to deploy space spraying within a radius of 1 city block from the block where each case occurred, which means such application likely had a negligible impact on the entomological measures in our study (Fig. S2).

**Figure 3:**
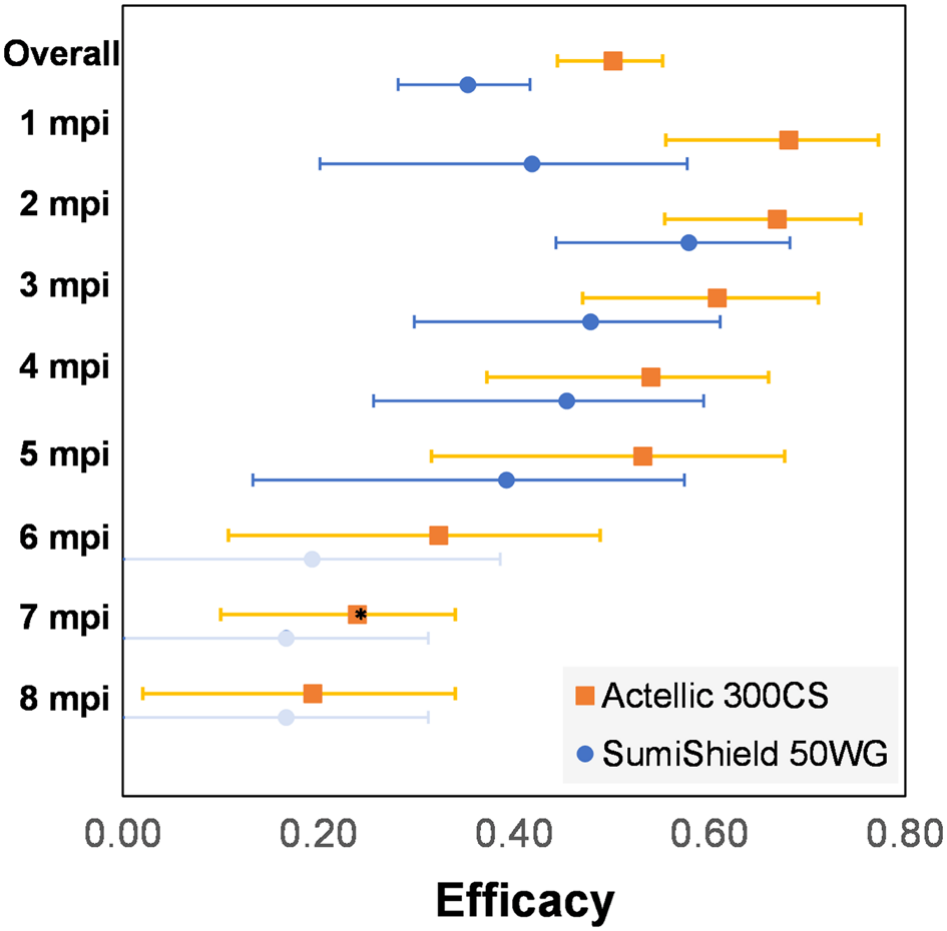
Estimated intervention efficacy. Expressed as the proportional reduction compared to the control arm in reducing *Ae. aegypti* density per house (squares and circles) and the predicted 95%CI of such estimates (error bars). Efficacy was calculated for the overall effect (during the 8 months post-intervention, mpi) and for each mpi. Solid colors show significant efficacy compared to the control, grayed colors non-significant associations and * marginal (P < 0.1) associations (see Table S4).

An overall (throughout the 8-month evaluation) statistically significant reduction in *Ae. aegypti* density compared to the control after pre-season TIRS was quantified for both TIRS arms (Table S3). When broken-down by MPI, Actellic 300CS significantly reduced *Ae. aegypti* density up to 8 months, whereas SumiShield 50WG up to 5 months (Table S3). The overall (throughout the 8-month evaluation) estimated intervention efficacy in reducing *Ae. aegypti* density was 50% (95%CI, 44–55%) for Actellic 300CS and 35% (28–41%) for SumiShield 50WG (Fig. 3). If only considering the period of peak ABV transmission (August-November) efficacy increased to 56.2% (46.8–63.9%) and 43.3 (31.4–52.9%), respectively. Broken down by MPI, there was no significant difference in efficacy between Actellic 300CS and SumiShield 50WG (Fig. 3). A similar trend in efficacy was quantified for density of bloodfed females (Table S4).

**Figure 4:**
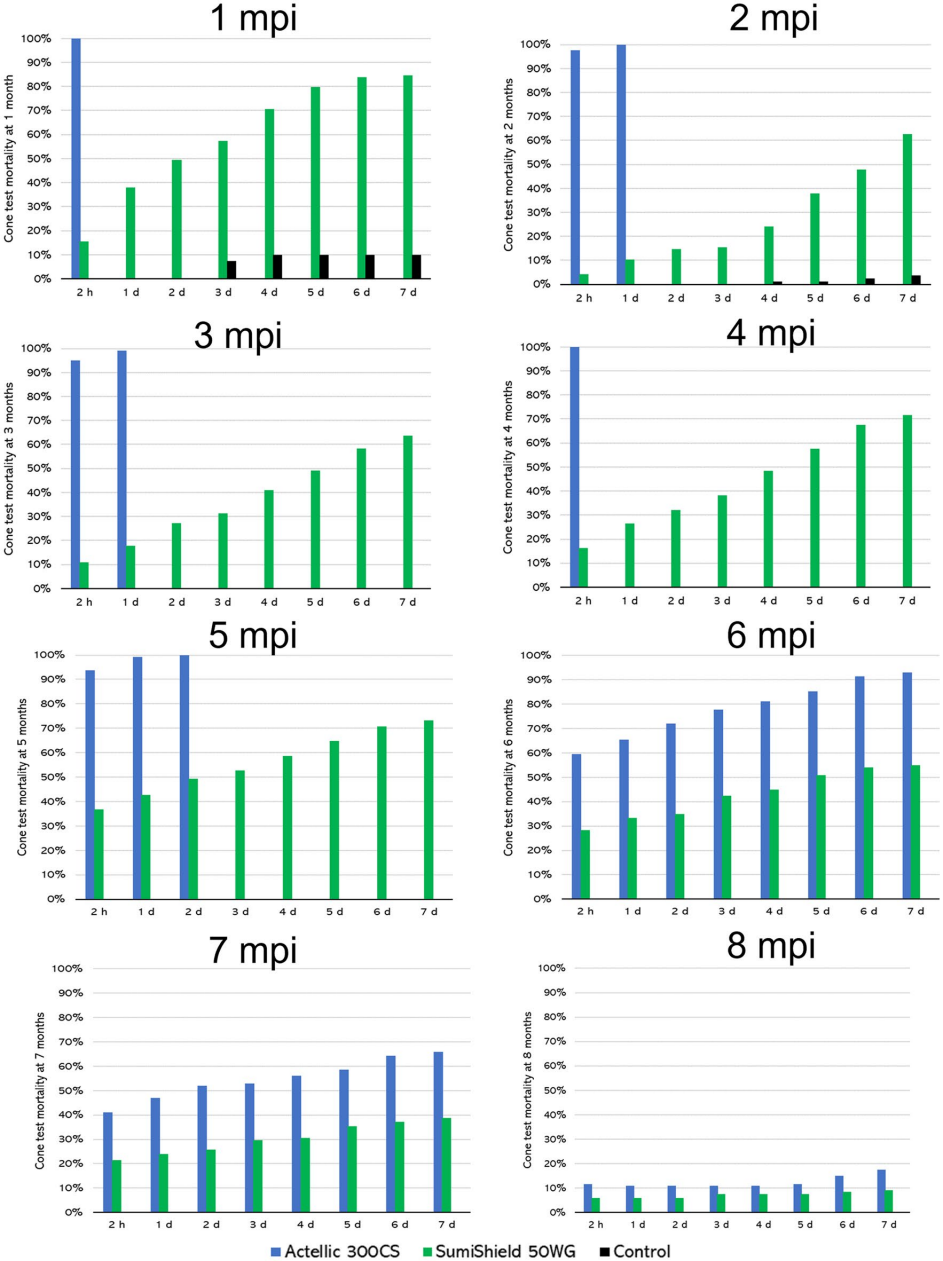
World Health Organization cone bioassays. Conducted up to 8 months post-intervention (mpi) using the Rockefeller *Ae. aegypti* strain. The graphs show acute (2 h after the 30-min exposure in the cones) and delayed (1 day to 7 days after the 30-min exposure in the cones) mortality values for each mpi.

WHO cone bioassays confirmed the entomological residual impact of both insecticides (Fig. 4). Actellic 300CS showed strong acute mortality (> 80%) up to 5 MPI, and up to 7 MPI when delayed mortality was assessed (Fig. 4). For SumiShield 50WG, acute mortality was very low (< 40%) throughout the evaluation, but when delayed mortality was factored in, mortality was higher than 80% only during the first month post-spraying (Fig. 4). Knock-down values after exposure to different active ingredients in CDC bottle bioassays were 72%, 94% for the pyrethroids permethrin and deltamethrin, and 100% for the organophosphate chlorpyrifos (Fig. S3).

Over 98% of participants interviewed at 3 MPI mentioned they would recommend TIRS with either insecticide (Table 1). While there were no reported concerns with the mode of application (duration, presence of personnel), respondents identified that Actellic 300CS had more smell than SumiShield 50WG (39.5% vs 4.3%) and left more stains in walls (8.6% vs 1.5%) (Table 1). Staining of walls with Actellic 300CS led 6.2% of respondents to clean the treated surfaces with a washcloth. Overall, 94–99% of respondents indicated no health impacts in them or a member of their family after insecticide application (the few health effects of the insecticide indicated transient effects such as headache, sneezing or eye irritation). More than half respondents perceived a reduction in mosquitoes indoors after the application (Table 1).

**Table 1:** Social perception and acceptability of preventive TIRS.

Question	Answer	Actellic 300CS (N & %)		SumiShield 50WG (N & %)	
Would you recommend TIRS?	No	1	1.3	0	0.0
	Yes	79	98.8	66	100.0
Discomfort with mode of application? (moving furniture, spray walls)	No	81	100.0	68	100.0
	Yes	0	0.0	0	0.0
Smell of insecticide?	Too much	3	3.7	0	0.0
	A lot	6	7.4	0	0.0
	A little	23	28.4	3	4.3
	None	49	60.5	65	94.2
Staining in walls?	Too much	0	0.0	1	1.5
	A lot	3	3.7	0	0.0
	A little	4	4.9	0	0.0
	None	74	91.4	67	98.5
Cleaning surfaces after spraying?	Too much	0	0.0	0	0.0
	A lot	3	3.7	1	1.5
	A little	2	2.5	0	0.0
	None	76	93.8	67	98.5
Discomfort with duration of application?	No	81	100.0	67	98.5
	Yes	0	0.0	1	1.5
Any health problems associated with the intervention?	No	75	93.8	66	98.5
	Si	5^a^	6.3	1^b^	1.5
Did you perceive any impact on mosquitoes compared to prior of the intervention?	No	21	26.3	9	13.4
	Less	48	60.0	38	56.7
	Same	9	11.3	12	17.9
	More	2	2.5	8	11.9

Surveys conducted in 150 houses receiving either Actellic 300 CS (N = 81) or SumiShield 50 WG (N = 68) and conducted 3 mpi (July 2019).

^a^1 headache, 1 eye irritation, 3 flu-like symptoms.

^b^Sneezing due to smell.

Insecticides represented approximately 82.8% of total costs per house of TIRS, considering the amount Mexico’s MOH pays for Actellic 300CS (US$54/bottle) (Table 2). Under this costing scenario, preventively treating a house and protecting it throughout the ABV transmission season with TIRS would cost $10.5 (Table 2). We additionally considered a scenario using the price of insecticides paid by the Global Fund for malaria control (US$15 per bottle or sachet)^36^. Under such scenario, insecticides constituted 57.3% of total costs and cost of preventive TIRS per house was reduced to US$4.2 (Table 2).

**Table 2:** Estimated costs of implementing preventive TIRS in Merida, Mexico.

Item	Category	Description^a^	Unit cost US$ (per day)^a^	Cost per house^b^
Personnel	Personnel	Considering a monthly salary of MX$6300	$9.77	$0.49
	PPE	Overalls, boots, gloves and mask delivered 2 × year MX$21,823.95/year	$2.78	$0.14
Spraying equipment	Sprayers	IK-vector costs MX$10,100 and equipment lasts 4 years	$0.32	$0.02
	Repair kits for sprayers	Sprayers should be repaired 1xyear, cost of kit for repairs MX$3,061.11	$0.39	$0.02
Vehicles	Vehicles	Pick-up truck costs MX$532,300 and has a typical life of 12 years in operation	$5.66	$0.28
	Vehicle maintenance	Vehicle maintenance performed 2 × year for MX$55,000 per year	$7.01	$0.35
	Gasoline	Approx. 10 L per day per vehicle at a cost of MX$21.99 per L	$10.23	$0.51
Insecticide	Insecticide	One bottle/sachet mixed in 7.5 L IK-vector sprayer. Per house a team consumes 1.214 L of diluted insecticide^b^		$8.64^c^
Total cost				$10.45^d^
Cost excluding equipment (vehicles and sprayers)				$9.87^c^
Proportional cost of insecticides (% of total cost)				82.80%

^a^Considering a 2020 average exchange rate of MX$21.49 per 1 US$.

^b^Assuming an average efficiency of 18 houses/day per team.

^c^Considering the amount paid by Mexico’s MOH for Actellic 300CS (US$54/bottle) in 2018, and using the following equation: (US$54*1.2 L)/7.5 L =.

^d^Assuming a cost of insecticide of $15 per bottle (Global Fund cost of Actellic 300CS and SumiShield 50WG)^36^ total costs per house would be $4.23 (and $3.57 if equipment costs are excluded).

## Discussion

Conducting a single TIRS application, averaging ~ 9 min per house (compared to ~ 25 min per house for classic IRS), led to sustained and significant reductions in *Ae. aegypti* that extended throughout the entire ABV transmission season. Compared to reactive peridomestic application of ephemeral ULV space spraying, preventive TIRS has multiple entomological benefits. Since a large number of *Ae. aegypti* rest indoors and in specific locations within the house^23^, TIRS maximized insecticidal impact. Moreover, the long residual duration quantified for both insecticides (up to 5–7 months) provided full-season protection by maintaining *Ae. aegypti* densities to levels comparable to the low ABV transmission season in the evaluated year. While TIRS may appear intrusive to the community, the benefits seen by householders make it desirable and worth recommending others. Costs per house were primarily driven by the cost of insecticides, which could provide an opportunity for significant cost-savings if prices were similar to those for malaria control.

The implementation of long-lasting interventions to prevent vector-borne pathogen transmission has been a common practice in the Americas and worldwide^37^, with periodic traditional IRS routinely used to control Chagas’ disease and Leishmaniasis^37^ and traditional IRS plus long-lasting insecticide-treated nets to prevent malaria transmission^38^. While contemporary *Ae. aegypti* control has relied heavily on peridomestic space spraying either in reaction to cases or preventively in response to increased numbers of *Ae. aegypti*, early efforts to control yellow fever relied on residual perifocal spraying^19^. The large extension and complexity of urban areas has led country ABV control agencies to focus efforts on the prevention of explosive epidemics by implementing peridomestic spraying (either thermal fogging or ULV)^9,39^. Extensive evidence shows such approach has limited entomological and epidemiological impact^40,41^. Indoor space spraying has shown higher entomological and epidemiological impact than peridomestic spraying^41,42^, yet its effect is short-lived. Capitalizing on *Ae. aegypti* behavior and the seasonality of ABV transmission, preventive TIRS may be incorporated into IVM plans as a complementary approach to mosquito space spraying and source reduction^43^.

In most of the tropics, and particularly in the Americas, ABVs are transmitted seasonally, coinciding with the rainy season^44^. Vector control activities tend to also be concentrated during the same period. Thus, conducting preventive (pre-season) TIRS would not compete with other vector control activities and would provide an efficient approach for intervention delivery. A recent WHO-PAHO manual was developed to aid MOHs in incorporating TIRS within their IVM plans^24^. Another component that can be incorporated to efficiently deploy TIRS involves capitalizing on the high spatial heterogeneity in ABV transmission within cities, in which some neighborhoods concentrate a high burden of disease compared to the entire city^26,30^. The PAHO has taken such evidence and turned it into a new IVM framework that utilizes spatial analysis and public health information to stratify urban areas based on arbovirus transmission risk^45^. Risk stratification under this novel IVM PAHO framework is based on the application of spatial clustering tests (Getis Gi* hotspot analysis) to the number of cases per urban census tract to identify tracts with significantly higher cases than predicted by chance^1^ By stratifying urban areas according to their risk of ABV transmission, preventive interventions such as TIRS could be implemented in ‘high risk’ areas to reduce transmission while optimizing limited human and economic resources.

Insecticide resistance is a major threat to the efficacy of existing *Ae. aegypti* control efforts^13,27^. Pyrethroid resistance is dominant in the Americas^13^ and, while results from CDC bottle bioassays may not always imply that a specific pyrethroid formulation will not be efficacious in field conditions, there is a need for the development of insecticides to which mosquitoes are fully susceptible. Efforts to mitigate insecticide resistance in malaria vectors has led to next generation insecticide formulations such as Actellic 300CS and SumiShield 50 WG^28,38^. In Africa, incorporating Actellic 300CS in nationwide malaria control programs led to significant reductions in malaria cases and *Anopheles* spp. numbers^46^. SumiShield 50 WG has been recently evaluated in trials, showing important reductions of *Anopheles* spp. entomological indices^47^. Alternative formulations using clothianidin exist, however these also contain pyrethroids which are already highly resisted by *Aedes* mosquitoes (Fludora Fusion, Bayer, clothianidin + deltamethrin), but do still appear to be effective in areas where pyrethroid resistance is prevalent.Our study is the first field trial of both insecticides against field (pyrethroid resistant) *Ae. aegypti* and provides evidence of non-pyrethroid active ingredients with high potential for preventive control. While Actellic 300CS had slightly higher efficacy and longer residual effect, the community had more negative reactions to it (due to smell and staining of walls) than SumiShield 50 WG. Our findings point to an important factor in the development of new residual insecticide formulations for urban areas: the need for maximizing both entomological efficacy and community acceptability of the insecticide and its application.

The control and both insecticide treatment arms showed a decreased trend in *Ae. aegypti* density during the month predicted as the peak of ABV transmission. While our study team lacked entomological information to determine whether 2018 was a ‘typical’ year with regards to *Ae. aegypti* numbers, we can attribute such reduction in the control to factors other than our study intervention. The low number of cases reported in southern Merida (only 18 cases, Fig S2) was an indicator that 2018 was not a high transmission year. Too much rain can impact negatively mosquito populations^48^. In 2018, Hurricane Michael formed in the Caribbean increasing the frequency and amount of rainfall in September–October in the Yucatan peninsula. The increased rainfall from those storms may have impacted *Ae. aegypti* numbers in the period of predicted peak dengue activity. Other factors, such as increased household use of insecticides (which can have an effect on *Ae. aegypti*) after storms due to the invasion by salt marsh *Aedes taeniorhynchus* mosquitoes, cannot be ruled out. Despite these effects, all preventive TIRS treatments led to a significant reduction in *Ae. aegypti* indices compared to the control arm.

In our study, WHO cone bioassays showed poor residual efficacy of SumiShield 50WG, yet entomological indicators showed significant reductions over a 5 month period. This mismatch may be explained by clothianidin’s unique mode of action. A recent study conducted by our team using *Ae. aegypti* from the trial site showed that cone bioassays provide poor results in surfaces treated with SumiShield 50 WG compared to the insecticide's impact on free-flying mosquitoes (released in experimental houses). While delayed mortality in cones reached a maximum of 60% at 1 month post-spraying, delayed mortalities higher than 80% were maintained up to 7 months when mosquitoes were exposed in experimental houses^49^. This difference may be the product of the insecticide mode of action. Clothianidin targets the nicotinic acetylcholine receptor (nAChR) in the insect central nervous system^50^, leading to a delayed effect on the mortality of mosquitoes. As seen with other insecticide, chlorfenapyr^51^, clothianidin increases its efficacy when mosquitoes are free-flying.

A recent study, conducted in towns surrounding Merida, estimated a weekly cost of releasing *Wolbachia*-carrying male *Ae. aegypti* (with the *w*AlbB strain) for population suppression of US$403.8 (or US$8.1 per hectare, or US$0.4 per house)^52^. Excluding infrastructure costs required to produce mosquitoes, *Wolbachia*-based population suppression would cost US$1.6 per house/month, or US$9.6/house to cover a 6-month transmission season. The same study estimated that a single round of peridomestic ULV using Malathion would cost US$863.8 per 50-hectares^52^, or (assuming 20 houses per block) US$0.86 per house. To have meaningful entomological impact, ULV has to be performed in multiple weekly cycles (a minimum of 4)^53^, which means costs per month of ULV would be around $3.44 per house, or $20.6 per house for the 6-month transmission season. Therefore, the cost of preventive TIRS at the cost of insecticides for Mexico’s MOH (US$ 10.5) would make this intervention competitive to existing and novel approaches. As options for residual insecticides in urban areas broaden, prices for MOHs in the Americas should lower, leading to lower TIRS intervention costs. Our estimate (using Global Fund costs of insecticides^36^) of US$4.2 per house would make TIRS much more appealing option for MOHs.

A Phase-III clinical trial ongoing in Merida, Mexico, is quantifying the epidemiological impact of preventive TIRS on ABV illness and infection in a cohort of 4600 children^25^. Results from such study will provide an opportunity not only to quantify the efficacy of preventive control interventions, but also an avenue to quantify the cost-effectiveness of this approach under different levels of insecticide coverage and entomological efficacy. The global context of ABV transmission requires innovative approaches that are effective and scalable. Conducting preventive residual insecticide applications can maintain *Ae. aegypti* densities at low levels year-round with important implications for preventing dengue, chikungunya and Zika epidemics.

## Supplementary Information

Supplementary Information.

## Data Availability

The datasets analyzed during the current study are available in the MendeleyData repository, Mendeley Data, V1, 10.17632/d7ft5vdwhr.1. https://data.mendeley.com/datasets/d7ft5vdwhr.
