# Supplementary material for: Preventive residual insecticide applications successfully controlled Aedes aegypti in Yucatan, Mexico
Source: Sci Rep. 2022 Dec 20;12:21998. doi: 10.1038/s41598-022-26577-1 (PMC9768150; doi:10.1038/s41598-022-26577-1)

Supplementary Figures and Tables

Preventive residual insecticide applications successfully controlled *Aedes aegypti* in Yucatan, Mexico

Gonzalo M Vazquez-Prokopec [1*]
Azael Che-Mendoza[2]
Oscar D. Kirstein [1]
Wilberth Bibiano-Marin[2]
Gabriela Gonzalez-Olvera [2],
Anuar Medina-Barreiro [2]
Hector Gomez-Dantes [3]
Norma Pavia-Ruz [4]
Pablo Manrique-Saide [2]

1. Department of Environmental Sciences, Emory University, Mathematics and Science Center, 400 Dowman Drive Ste: E530, Atlanta, GA 30322, USA
2. Unidad Colaborativa para Bioensayos Entomologicos, Universidad Autonoma de Yucatan, Mexico
3. National Institute of Public Health, INSP, Cuernavaca, Mexico.
4. Centro de Investigaciones Regionales, Autonomous University of Yucatan, Merida, Mexico.

**Correspondence to:*
Gonzalo Vazquez-Prokopec.
Email: gmvazqu@emory.edu

Table S1. Schedule of activities throughout the trial and according to the seasonality of *Aedes*-borne viruses (ABV).

**Activities**	**Date**	**ABV season**
Baseline	April 10-23, 2018	Low
**TIRS intervention**	**June 17-26, 2018**	**Low**
1 month	July 22-August 1, 2018	Medium
2 months	August 19-29, 2018	High
3 months	September 17-26, 2018	High
4 months	October 21-28, 2018	High
5 months	November 18-29, 2018	High
6 months	December 16-23, 2018	Medium
7 months	January 23-31, 2018	Medium
8 months	February 17-27, 2018	Low

Table S2. Main parameters for TIRS application in 500 houses of Merida, Mexico (Mean ± SD).

Formulation	Houses treated	Inhabitants / house	Rooms/ house	Rooms treated	Application time (min)/ house	Insecticide solution (ml)/ house	Surface (m^2) treated per house
Actellic 300CS	248	3.7± 1.7	4.4± 1.8	3.4±1.4	9.9± 5.1	1213.7± 782.7	40.5± 26.1
SumiShield 50WG	252	2.6± 2.1	4.8± 1.8	3.2±1.4	8.8± 4.5	955.9± 603.0	31.9± 20.1

Table S3. Results from a negative binomial GLMM showing the overall effect, the effect on the months when ABVs peak (September-November) and the monthly effect (expressed as months post-intervention, MPI) for the density of *Ae. aegypti* per house.

Effect	Parameter	Estimate	Std. Error	z	P-value
Overall (1-8 MPI)	Actellic 300CS	-0.6956	0.0547	-12.72	<0.001
	SumiShield 50WG	-0.435	0.053	-8.21	<0.001
Peak ABV (3-5 MPI)	Actellic 300CS	-0.8266	0.0986	-8.386	<0.001
	SumiShield 50WG	-0.5672	0.0962	-5.898	<0.001
1 MPI	Actellic 300CS	-1.1423	0.1707	-6.692	<0.001
	SumiShield 50WG	-0.5423	0.1616	-3.356	<0.001
2 MPI	Actellic 300CS	-1.1066	0.1519	-7.284	<0.001
	SumiShield 50WG	-0.865	0.143	-6.05	<0.001
3 MPI	Actellic 300CS	-0.9359	0.1536	-6.092	<0.001
	SumiShield 50WG	-0.6509	0.1491	-4.365	<0.001
4 MPI	Actellic 300CS	-0.777	0.1539	-5.049	<0.001
	SumiShield 50WG	-0.6052	0.1507	-4.017	<0.001
5 MPI	Actellic 300CS	-0.7587	0.1907	-3.979	<0.001
	SumiShield 50WG	-0.4981	0.1807	-2.757	0.0058
6 MPI	Actellic 300CS	-0.39	0.1412	-2.762	0.0057
	SumiShield 50WG	-0.2154	0.1379	-1.562	0.1182
7 MPI	Actellic 300CS	-0.2717	0.1514	-1.795	0.0726
	SumiShield 50WG	0.0322	0.1527	0.211	0.8329
8 MPI	Actellic 300CS	-0.2163	0.1006	-2.15	0.0316
	SumiShield 50WG	-0.183	0.0972	-1.883	0.0596

Table S4. Results from a negative binomial GLMM showing the overall effect, the effect on the months when ABVs peak (September-November) and the monthly effect (expressed as months post-intervention, MPI) for the density of bloodfed *Ae. aegypti* females per house. Efficacy (expressed as % reduction compared to the control) is shown with its 95% CI.

						Efficacy (%)		
		**Estimate**	**Std.Error**	**Z**	**P-value**	**Mean**	**Low 95%**	**High 95%**
Overall (1-8 MPI)	Actellic 300CS	-0.78875	0.06487	-12.159	<0.001	54.6	48.4	60.0
	SumiShield 50WG	-0.46053	0.06116	-7.53	<0.001	36.9	28.8	44.0
Peak (3-5 MPI)	Actellic 300CS	-0.78875	0.06487	-12.159	<0.001	54.6	48.4	60.0
	SumiShield 50WG	-0.46053	0.06116	-7.53	<0.001	36.9	28.8	44.0
1 MPI	Actellic 300CS	-1.2117	0.1797	-6.741	<0.001	70.2	57.9	79.2
	SumiShield 50WG	-0.562	0.1602	-3.508	<0.001	43.0	22.0	58.4
2 MPI	Actellic 300CS	-1.0071	0.1634	-6.165	<0.001	63.5	49.7	73.5
	SumiShield 50WG	-0.9193	0.1534	-5.992	<0.001	60.1	46.2	70.5
3 MPI	Actellic 300CS	-0.858	0.1837	-4.67	<0.001	57.6	39.8	70.8
	SumiShield 50WG	-0.5836	0.1715	-3.403	<0.001	44.2	22.0	60.5
4 MPI	Actellic 300CS	-0.68341	0.20728	-3.297	<0.001	49.2	29.4	70.0
	SumiShield 50WG	-0.39595	0.20102	-1.97	0.048873	34.2	8.4	51.6
5 MPI	Actellic 300CS	-0.7877	0.241276	-3.265	0.0011	55.6	49.4	60.0
	SumiShield 50WG	-0.53844	0.23158	-2.325	0.0201	43.0	26.7	52.8
6 MPI	Actellic 300CS	-0.63949	0.16482	-3.88	<0.001	47.2	27.4	62.0
	SumiShield 50WG	-0.42535	0.15584	-2.729	0.006347	34.6	11.5	52.0
7 MPI	Actellic 300CS	-0.40682	0.16213	-2.509	0.0121	33.4	8.4	51.6
	SumiShield 50WG	-0.04116	0.15542	-0.265	0.7911	0.4	0.3	29.1
8 MPI	Actellic 300CS	-0.51086	0.13087	-3.904	<0.001	40.0	22.7	53.8
	SumiShield 50WG	-0.1828	0.12015	-1.521	0.128155	16.8	0.5	34.3

Figure S1. Impact of pre-season TIRS on bloodfed *Ae. aegypti* density. Estimated mean (±95%CI) number of *Ae. aegypti* females with evidence of a recent bloodmeal per house at baseline and 1-8 months post-TIRS application of two long-lasting residual insecticide formulations in Merida, Mexico.

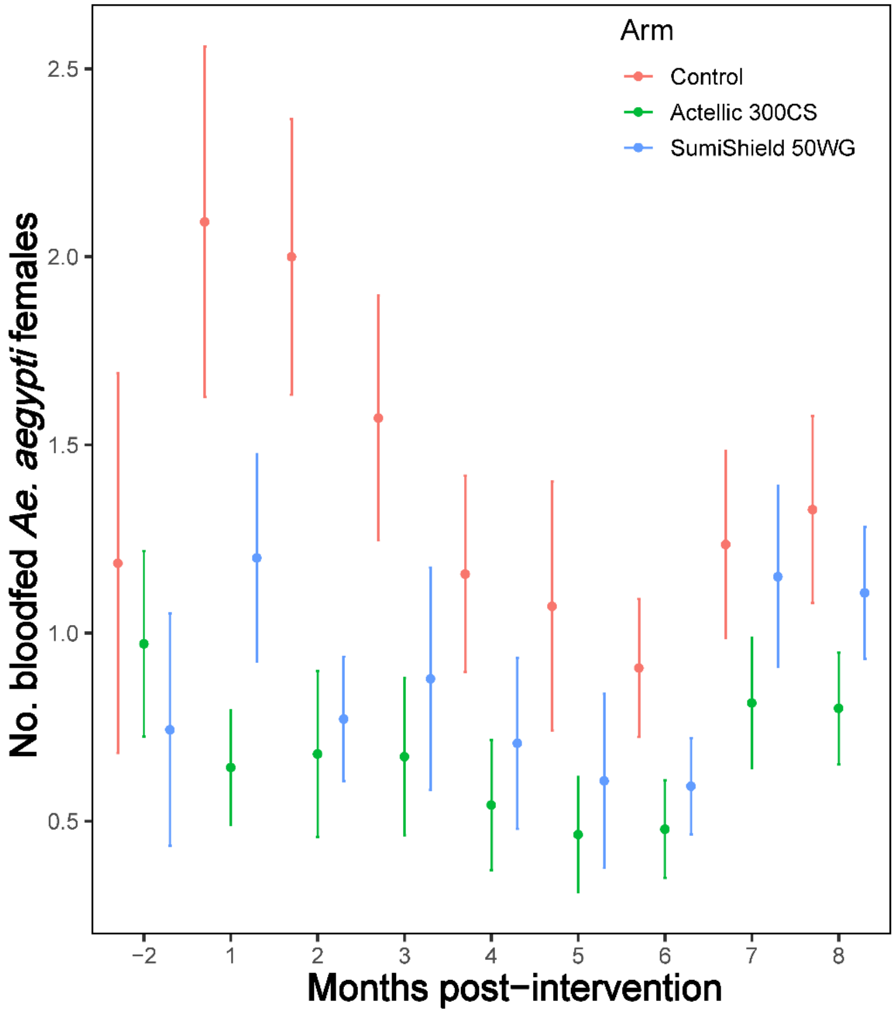

Figure S2. Distribution of confirmed dengue virus cases (points) reported by the Ministry of Health in southern Merida, 2018. None of the cases occurred within the study clusters, and vector control actions were focused within one the city block around the block where the cases occurred, as no outbreak was declared to justify city-wide ULV.

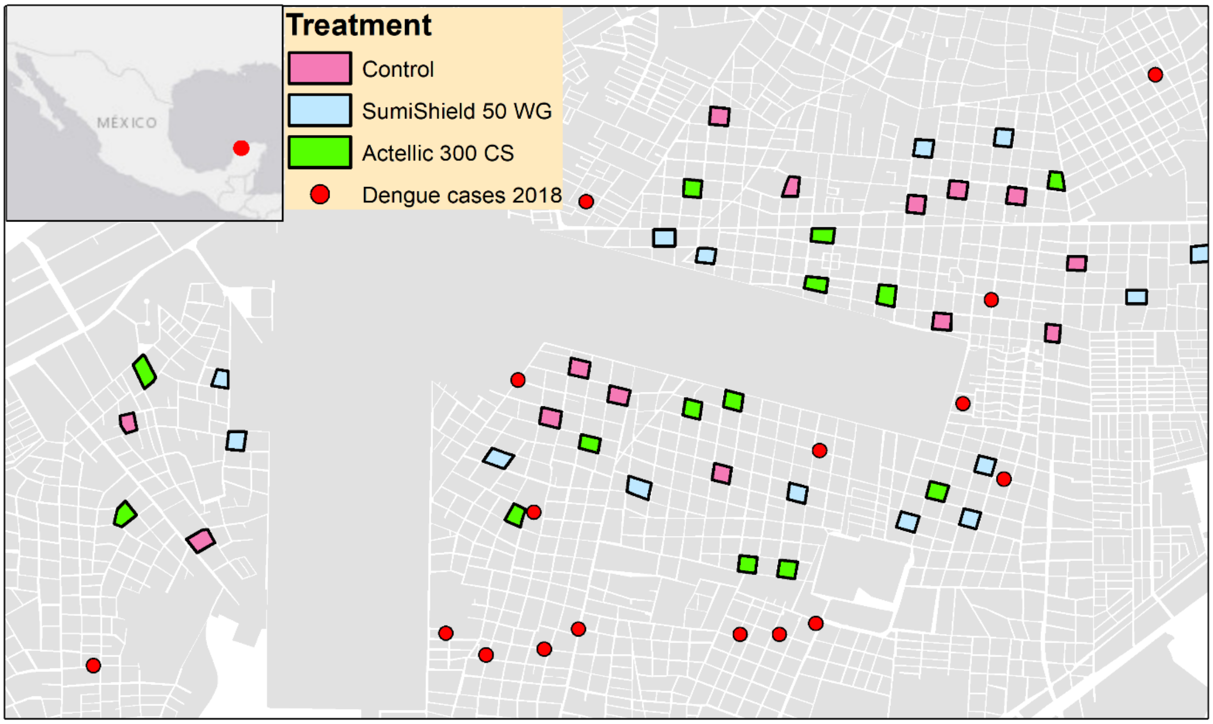

Figure S3. Percent knockdown of female *Ae. aegypti* at diagnostic time (30 mins) when exposed to diagnostic dose of permethrin, deltamethrin and chlorpyrifos.

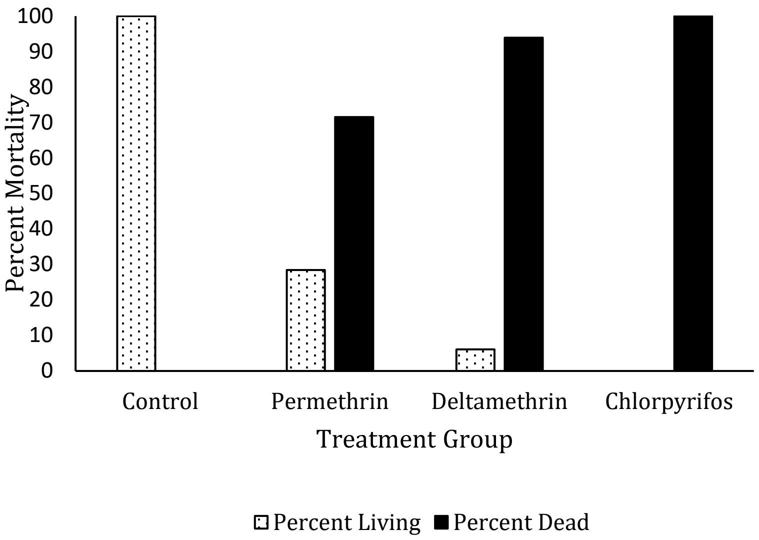

Supplement: Supplementary file 1 — Supplementary Information. [file 41598_2022_26577_MOESM1_ESM.pdf]
